# *Plagiotheciumschofieldii*, a new species from the Aleutian Islands (Alaska, USA)

**DOI:** 10.3897/phytokeys.184.69970

**Published:** 2021-11-05

**Authors:** Grzegorz J. Wolski, Paulina Nowicka-Krawczyk, William R. Buck

**Affiliations:** 1 Department of Geobotany and Plant Ecology, Faculty of Biology and Environmental Protection, University of Lodz, ul. Banacha 12/16, 90-237 Lodz, Poland; 2 Department of Algology and Mycology, Faculty of Biology and Environmental Protection, University of Lodz, ul. Banacha 12/16, 90-237 Lodz, Poland; 3 Institute of Systematic Botany, The New York Botanical Garden, Bronx, NY 10458-5126, USA

**Keywords:** Bryophyta, Plagiotheciaceae, taxonomy, W. B. Schofield

## Abstract

*Plagiotheciumschofieldii***sp. nov.** is described from the Aleutian Islands, Alaska, U.S.A. Some morphological features of this species correspond to *P.lamprostachys* (Southern Hemisphere species); however, *Plagiotheciumschofieldii* is genetically and morphologically different from this and other common Northern Hemisphere species e.g., *P.denticulatum*, *P.platyphyllum*, or *P.ruthei*. The most important distinguishing morphological features differentiating this species are: the arrangement of the leaves on the stem; dimensions, concavity and symmetry of the leaves; dimensions of cells and their areolation; orientation of capsules. Additionally, due to the strong concavity of the leaves, they are very often badly damaged under the microscope. We present the results of DNA research of the analyzed samples, and a detailed description of the morphological features. The new species is illustrated, and its ecological preferences and currently known geographical distribution are presented. Additionally, the authors propose to add this species to *Plagiothecium* section, which is confirmed by morphological features and genetic analysis.

## Introduction

Over the last several years, our perception has changed not only of *Plagiothecium* Schimp., but also of the whole family of Plagiotheciaceae M.Fleisch. (e.g., Pedersen and Hedenäs 2001, 2002; Wynns et al. 2018). The use of molecular methods has not only helped to understand many taxa previously considered problematic, but has also allowed for the description of a number of new taxa (e.g., Zuo et al. 2011; Wynns et al. 2018; Ignatova et al. 2019; Wolski and Nowicka-Krawczyk 2020). Nevertheless, for decades the taxonomic status of many species of this genus has been unclear and ambiguous, and those taxa currently require detailed morphological, genetic and taxonomic studies.

Although the Northern Hemisphere seems to be relatively well researched, there are still many areas (e.g., central Asia, Middle East) which remain as gaps on the world distribution map of *Plagiothecium* (Wolski et al. 2021). The results of taxonomic revisions conducted in recent years indicate the underestimation of the species richness of individual parts of the world. As a consequence of this research, many countries and regions have increased their number of ​known taxa of the described genus (e.g., Ellis et al. 2019a; Ellis et al. 2019b; Ellis et al. 2020, 2021; Müller and Wynns 2020; Wolski and Nowicka-Krawczyk 2020; Wolski 2020).

The Aleutian Islands, Alaska, U.S.A., are one of the many under-explored regions of the Northern Hemisphere. As a result of the taxonomic revision of *Plagiothecium* specimens from this area it was possible to describe a new species from this genus; the results are presented below.

## Materials and methods

### Taxonomic analyses

Material from the Missouri Botanical Garden (MO), The New York Botanical Garden (NY) and the University of British Columbia (UBC) was analyzed during the revision of *Plagiothecium* from the Aleutian Islands. For selected specimens intended for DNA analysis, appropriate consent was obtained from NY (NY02589541) and MO (MO5135779, MO5140205, MO5148015).

### DNA isolation, amplification and sequencing

The molecular research was based on nuclear and chloroplast DNA markers: ITS (from the 3’ end of the hypervariable nuclear spacer ITS1, through the 5.8S gDNA, to the 5` end of the ITS2 spacer); and *rpl16* cpDNA gene encoding ribosomal protein L16. Markers were selected based on Wynns et al. (2018), Ignatova et al. (2019) and Wolski and Nowicka-Krawczyk (2020) from *Plagiothecium*-focused studies.

Leafy stems of mosses were cut from dried material. Approximately 20 mg of dry tissue from each specimen in duplicates was placed in a 1.5 ml Eppendorf Safe-Lock tube and frozen (-20 °C) for homogenization. Tissue homogenization was performed using a hand-held stainless steel homogenizer (Schlüter Biologie, Eutin, Germany). Total DNA was extracted using the GeneMATRIX Plant & Fungi DNA Purification Kit (Eurx, Gdansk, Poland) following the manufacturer’s protocol. DNA extracts were quantified with a BioDrop DUO Spectrophotometer (BioDrop Ltd, Cambridge, U.K.). From the duplicates, the sample with the higher quality DNA (1.7–1.9 OD_260_/OD_280_) was selected for further analysis.

For each sample, all markers were amplified by PCR in a few replicates to obtain high quality amplicons for sequencing. PCR was performed using primers and reaction conditions as described in Wolski and Nowicka-Krawczyk (2020), with a 50 µl reaction volume with 25 µl of Color Taq PCR Master Mix (2×) (Eurx, Gdansk, Poland).

PCR products were visualized on an agarose gel (1.5%, 90V, 40 minutes) stained with GelRED fluorescent dye (Biotum, Fremont, CA, U.S.A.) and two replicates of each marker per sample were chosen for sequencing. Amplicons from the PCR reaction were cleaned using Syngen Gel/PCR Mini Kit (Syngen Biotech, Wrocław, Poland) according to the manufacturer’s protocol. Samples were sequenced with Sanger sequencing using primers from amplification by SEQme s.r.o. company (Dobris, Czech Republic). The obtained sequences were assembled in Geneious 11.1.5 (Biomatters Aps, Aarhus, Denmark) (http://www.geneious.com). The sequences were submitted to the NCBI GenBank database (www.ncbi.nlm.nih.gov) under the accession numbers MW936654- MW936657 for ITS and MW935831–MW935834 for *rpl16*.

### Phylogenetic analyses

Phylogenetic analyses of studied specimens and other species in the *Plagiothecium* group were performed based on a concatenated ITS-*rpl16* sequence matrix. Voucher information for the specimens included in this study, with corresponding GenBank accession numbers, is presented in Table 1. Sequences were aligned using the MAFFT v. 7 web server (Katoh et al. 2017) (http://mafft.cbrc.jp/alignment/server/) where the auto strategy was applied, the scoring matrix of 200PAM with Gap opening penalty of 1.53, UniREf50 for Maft-homologs and Plot and alignment with threshold of 39 score were set. The obtained alignments were checked for poorly and ambiguously aligned regions and small corrections were made by eye. The evolutionary models were calculated using PartitionFinder 2 software (Lanfear et al. 2016) chosen according to the Akaike Information Criterion (Table 2).

**Table 1.** Voucher information and accession numbers for the specimens included in the phylogenetic analyses.

Taxon	Collection	Locality	ITS	rpl16
*Plagiotheciumberggrenianum*	S-B44769	Russia: Pacific Siberia, Yakutiya	KY550267	KY513972
*Plagiotheciumbrasiliense*	E barcode E00387968	Brazil	KY550266	KY513971
*Plagiotheciumconostegium*	NY: *S.P. Churchill* et al. *19839*	Bolivia	KY550271	KY513976
	NY barcode 00845279	Guatemala	KY550318	KY514024
	S-B53327	Mexico	KY550272	KY513977
*Plagiotheciumcurvifolium*	DUKE barcode 0209096	Canada: BC	KY550273	KY513978
	CP: *G.P. Rothero s.n.*	Germany: Hochschwarzwald	KF882228	KF882328
*Plagiotheciumdenticulatum*	CP: *J.T. Wynns 2081*	Denmark: Sorø kommune, Sj*æ*lland	KF882229	KF882329
Plagiotheciumdenticulatum var. bullulae	UC barcode 1947417	USA: CA	KY550277	KY513982
	UC barcode 1798690	USA: NV	KY550278	KY513983
Plagiotheciumdenticulatumvar.obtusifolium	CP: *J.T. Wynns 2842*	Germany: Schauinsland, Hochschwarzwald	KF882230	KF882330
	UC barcode 1724036	USA: WA	KY550279	KY513984
Plagiotheciumdenticulatum fo. pungens	DUKE barcode 0150010	USA: AK	KY550280	KY513985
*Plagiotheciumlaetum*	CP: *J.T. Wynns 2907*	Germany: Schauinsland, Hochschwarzwald	KF882234	KF882334
	C barcode CP0010626	USA: NC	KY550292	KY513997
	C barcode CP0010627	USA: NC	KY550293	KY513998
	OK2066	Germany	MK934644	MK941642
	OK2035	Russia: Krasnodar, Shakhe	MK934647	MK941645
*Plagiotheciumlamprostachys*	S-B54613	Australia: VIC	KY550284	KY513989
	DUKE barcode 0156846	Australia: VIC	KY550285	KY513990
*Plagiotheciumlatebricola*	CP: *I.L. Goldberg s.n.*	Denmark: Holmegårds Mose, Sj*æ*lland	KF882235	KF882235
*Plagiotheciumlucidum*	NY barcode 01233548	Chile	KY550298	KY514003
	BONN: *J.-P. Frahm 12–6*	New Zealand	KY550299	KY514004
*Plagiotheciummembranosulum*	BONN: *J.-P. Frahm 7756*	Democratic Republic of the Congo	KY550310	KY514015
	S barcode B78514	South Africa	KY550303	KY514008
	DUKE barcode 0016754	South Africa	KY550304	KY514009
*Plagiotheciummollicaule*	NY barcode 1596265	Brazil	KY550300	KY514005
*Plagiotheciumovalifolium*	DUKE barcode 0188886	Chile	KY550314	KY514019
*Plagiotheciumpacificum*	UC barcode 1921143	USA: CA	KY550295	KY514000
*Plagiotheciumplatyphyllum*	CP: *J. Lewinsky* et al. *s.n.*	Finland: Haluna, Nilsiae, Savonia borealis	KF882241	KF882341
*Plagiotheciumrossicum*	OIK-2019 isolate OK2054	Russia: Kunashir	MK934622	MK941625
	OIK-2019 isolate OK2032	Russia: Smolensk	MK934629	MK941630
*Plagiotheciumruthei*	CP: *J.T. Wynns 1997*	Denmark: Lyngby Aamose, Sj*æ*lland	KF882242	KF882342
*Plagiotheciumsvalbardense*	C-M-9109	Greenland: W5	KY550296	KY514001
*Plagiotheciumangusticellum*	*Wolski 22*	Poland	MN077507	MN311142
*Plagiotheciumlongisetum*	*Wolski 19*	Poland	MN077506	MN311141
*Isopterygiopsispulchella*	UC barcode 1947397	USA: CA	KY550336	KY514042
P1 MO5135779	MO5135779	USA: Alaska, Simeonof Island	MW936657	MW935834
P2 MO5140205	MO5140205	USA: Alaska, Simeonof Island	MW936656	MW935833
P3 MO5148015	MO5148015	USA: Alaska, Simeonof Island	MW936655	MW935832
P4 NY02589541	NY02589541	USA: Alaska, Adak Island	MW936654	MW935831

**Table 2.** Summary of partitions for ITS-*rpl16* matrix (1574 bp) evolutionary model selection and phylogenetic interference using PartitionFinder2.

	ITS1	5.8S gDNA	ITS2	*rpl*16 intron	rpl16 codon
ML	JC	JC	HKY +I	TIM+I+G	JC
BI	JC	JC	HKY	F81	JC

Phylogenetic calculations were performed using maximum likelihood analysis (ML) in the IQ-TREE web server (Trifinopoulos et al. 2016) (http://iqtree.cibiv.univie.ac.at/) with the ultrafast bootstrap (UFBoot) pseudo likelihood algorithm (Hoang et al. 2018) and 10000 replicates; and Bayesian inference (BI) in MrBayes 3.2.2 (Ronquist et al. 2012) where two parallel Markov chain Monte Carlo (MCMC) runs for four million generations each, with trees sampled every 1000 generations. The average standard deviation of split frequencies in both cases remained below 0.01 for the last 1000 generations and posterior probabilities were estimated from the 50% majority-rule consensus tree after elimination of the first 25% of samples as burn-in. Raw data sequences, the alignment and tree files were submitted to the figshare online database (https://doi.org/10.6084/m9.figshare.14443697.v1).

Haplotype network analysis was performed using Median Joining Network in PopART v. 1.7 with gap coding as a single event irrespective of length and haplotypes` geographic distribution (Leigh and Bryant 2015). The analysis included species of Plagiothecium sect. Plagiothecium with representatives of *P.longisetum* and *P.angusticellum* (sect. Orthophyllum).

## Results and discussion

**Figure 1.**
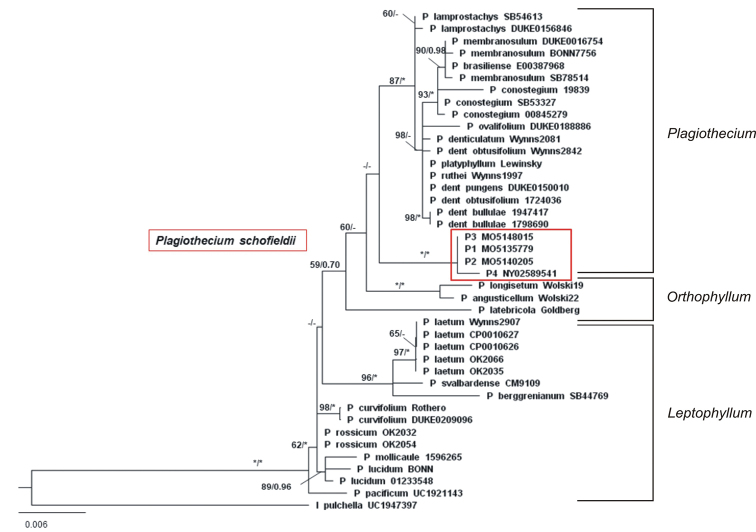
Phylogenetic tree of *Plagiothecium* taxa with *Isopterygiopsispulchella* as the outgroup based on concatenated nuclear (ITS1-5.8S-ITS2) and chloroplast (*rpl*16) DNA markers (total 1574 bp). The tree presents the position of *Plagiothecium* morphotypes from Alaska among the *Plagiothecium* group which is divided into individual sections. Numbers on branches indicate bootstrap values from ML followed by posterior probabilities from BI analysis. Asterisk (*) indicates 100 (ML) and 1.00 (BI), while minus (-) indicates values below 50 (ML) and 0.7 (BI). The topology of the tree was based on ML analysis.

Phylogenetic analyses based on the concatenated ITS-*rpl16* matrix placed studied specimens within the branch of a Plagiothecium sect. Plagiothecium clade, or sister to it; however, the branch support was very low (BS = 49). The next branch down is to representatives of sect. Orthophyllum Jedl. and even more distant to sect. Leptophyllum Jedl. clade (Fig. 1). After branching off from the Orthophyllum clade, the internal division of sect. Plagiothecium was well supported by Bayesian inference (PP ≥ 0.98). Maximal support from both maximum likelihood and Bayesian Inference was recorded in the clade of *Plagiotheciumschofieldii*, where the representatives create a monospecific clade (PP = 1).

**Figure 2.**
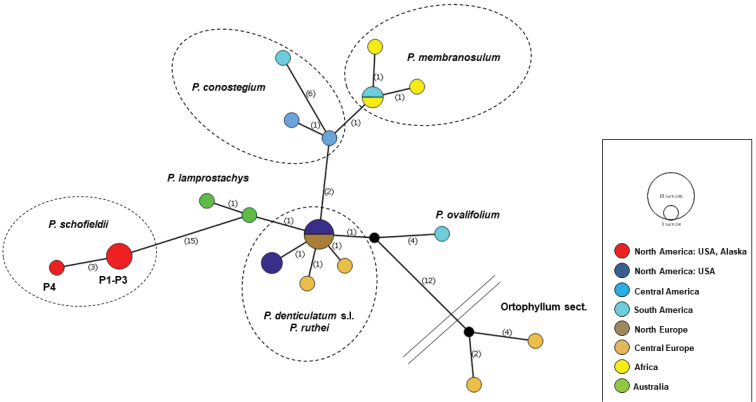
Median-joining haplotype network of sections *Plagiothecium* and *Orthophyllum* of *Plagiothecium* constructed in PopART. Haplotypes are represented by circles with colors indicating geographic distribution. The number on the branches indicates the mutational steps.

The haplotype network (Fig. 2) also showed internal diversity in sect.Plagiothecium. At the center, the analysis grouped haplotypes from the Northern Hemisphere (*P.denticulatum* and *P.ruthei*). Three branches extending from the center apply to haplotypes from Central America and the Southern Hemisphere. The position of *P.schofieldii* haplogroup is fairly isolated and consists of two haplotypes: the first refers to three specimens from Simeonof Island, while the second to a representative from Adak Island (Table 1); however, as it grows in the Aleutian Islands, Alaska, the branch vector points out the same direction as haplotypes from the Northern Hemisphere.

The individual taxonomic features of *Plagiothecium* are related to a specific level of detail in our analyses, and for example: superficial layer of the stem (epidermis layer) of large, thin-walled cells; shortly pointed leaves; serration (if present) only at apex; absence of pseudoparaphyllia; leaves clearly decurrent at the base – distinguish this genus from other genera belonging to the Plagiotheciaceae. Within *Plagiothecium*, the shape of decurrent alar regions, and the shape of their cells distinguishes the species of individual sections, while the shape and dimensions of leaf cells are the most important features distinguishing species from each other (Iwatsuki 1970; Lewinsky 1974; Noguchi 1994; Smith 2001). Therefore, based on the fact that the analyzed specimens have decurrent alar cells that are rounded, inflated, and form distinct auricles, as well as the shape and size of the leaf cells of *Plagiotheciumschofieldii*, we believe that this species belongs to Plagiotheciumsect. Plagiothecium. This is also confirmed by molecular and haplotype network analyses.

Species that are widespread in the Northern Hemisphere: *Plagiotheciumdenticulatum* (Hedw.) Schimp., *P.platyphyllum* Mönk., and *P.ruthei* Limpr., significantly differ in morphology from *P.schofieldii*, which, compared to the above-mentioned species, has erect stems, while the others are usually prostrate, or sometimes prostrate to ascending (Smith 2001; Li and Ireland 2008; Wynns 2015).

Leaves of *P.schofieldii* are julaceous and imbricate – very closely arranged on the stem, while in other species the leaves are strongly complanate, flaccid, and spreading on the stem. In the Northern Hemisphere only in *P.denticulatum* shoots are rarely julaceous (Lewinsky 1974; Smith 2001; Li and Ireland 2008; Wynns 2015). The appearance of the mats and the arrangement of the leaves on the stem are more similar to those features in *P.cavifolium* (Brid.) Z. Iwats. (which belongs to Plagiothecium sect. Orthophyllum).

Stem leaves of *Plagiotheciumschofieldii* are very strongly concave, to such an extent that under the microscope they are clearly damaged and cracked from being flattened by the coverslip. The leaves of the closely related species are rather flat. Only in the case of *P.denticulatum* are the leaves more or less concave, but never to such an extreme (Lewinsky 1974; Smith 2001; Li and Ireland 2008). *Plagiotheciumschofieldii* is characterized by symmetrical leaves, and from other members of sect. Plagiothecium only *P.platyphyllum* has more or less symmetrical leaves, but this is the only feature common to both species. Symmetrical leaves are typical, e.g., for species from sect. Orthophyllum (e.g., *P.nemorale*, *P.cavifolium*) (Lewinsky 1974; Smith 2001; Li and Ireland 2008; Wynns 2015; Wolski 2020). Also, leaves of *P.schofieldii* are clearly longer and wider than those of all the species mentioned above (Smith 2001; Li and Ireland 2008).

*Plagiotheciumschofieldii* is clearly distinguished from *P.denticulatum*, *P.platyphyllum* and *P.ruthei* by the length and width of it laminal cells. The cells located in the central part of the leaf are long and very wide (88–190 × 13–29 μm), which makes the cell areolation very loose. None of the above-mentioned species has such long and broad cells, and thus their cell areolation is tighter (Lewinsky 1974; Smith 2001; Li and Ireland 2008).

Another feature that clearly distinguishes this newly described species from the previous species in sect. Plagiothecium is the orientation of the capsules. In the studied specimens of *P.schofieldii*, the capsules are orientated most often more or less vertically, i.e., erect, rarely inclined. *Plagiotheciumdenticulatum*, *P.platyphyllum*, and *P.ruthei* have inclined capsules (Lewinsky 1974; Smith 2001; Li and Ireland 2008; Wynns 2015).

**Figure 3.**
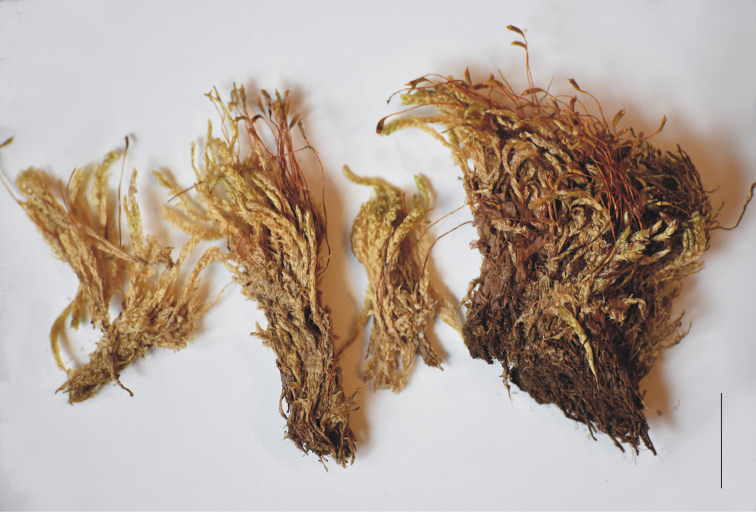
Stems with the sporophytes of *Plagiotheciumschofieldii*. Part of the turf of holotype (*W.B. Schofield 106119*, MO5135779). Scale bar: 1 cm.

**Figure 4.**
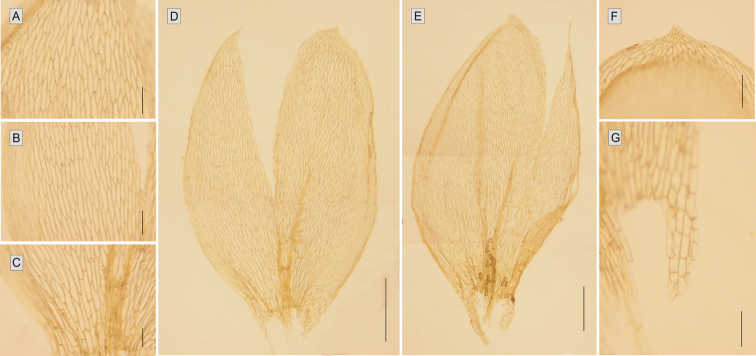
The most important taxonomic features of *Plagiotheciumschofieldii*. Dimensions of cells from the apex **A** the middle **B** and basal part of the leaf **C** leaves **D–E** leaf apex **F** auricles **G**. Scale bar: 100 µm (**A–C, F–G**); 500 µm (**D–E**). Photos from the holotype (*W.B. Schofield 106119*, MO5135779).

On the other hand, in terms of morphology, *P.schofieldii* looks more like *P.lamprostachys* (Hampe) A. Jaeger – a Southern Hemisphere species (Ireland 1992; Wynns 2015) – than the common Northern Hemisphere species mentioned above. Both the morphological features and molecular analyses indicate the distinctiveness of the species (Figs 1–4).

Additionally, Wynns (2015) pointed out that *P.lamprostachys* forms a clade within *P.denticulatumsensu lato*, which is also confirmed by our research (Fig. 1). Phylogenetic analyses of concatenated nuclear and chloroplast markers placed *P.schofieldii* within sect. Plagiothecium next to *P.denticulatum*; however, the branch support was very low. After branching off from the *Leptophyllum* clade (BI = 0.70), Bayesian inference highly supported the phylogenetic relations within sect. Plagiothecium indicating the separateness of a *P.schofieldii* clade (as well as a sect. Orthophyllum clade). This separation was also visible in the haplotype network, where the analysis extended the Alaskan clade far from the center of the network grouping species of *Plagiothecium* from the Northern Hemisphere.

All the above morphological data, supported by molecular studies, warrant the recognition of the Aleutian samples as a new species.

### Taxonomy

#### Plagiothecium schofieldii

Taxon classificationPlantaeHypnalesPlagiotheciaceae

G.J.Wolski & W.R.Buck sp. nov.

0B2FCA35-78CC-5069-8940-8E063D999603

##### Type.

U.S.A. Alaska, Shumagin Islands, Simeonof Island, mainly near saddle between Hill 1436 and 1265, wet cliff chimney, 54°55'N, 159°15'W, 19 July 1996, *W.B. Schofield 106119*, Holotype MO5135779.

##### Description.

Plants small, light green to yellow, with a delicate metallic luster, forming very dense, often homogeneous mats. Stems erect, 1.5–3.0 cm long (Fig. 3), in cross-section rounded, with a diameter of 300–380 μm, the central strand developed, epidermal cells 10–25 × 6–12 μm, the parenchyma thin-walled, 20–40 × 15–32 μm; leaves julaceous, imbricate, very closely arranged on the stem, when dry not shrunken, very concave, therefore very often damaged under the microscope, symmetrical, ovate to elliptic, those from the middle of the stem 1.4–3.0 mm long, and the width measured at the widest point 0.9–1.9 mm; the apex obtuse and apiculate, often denticulate; costae two, thick and strong, extending usually to ½ of the leaf length, reaching 0.5–2.0 mm; laminal cells linear, rather symmetrical, in quite regular transverse rows, the length and width very variable but dependent on location: 66–178 × 14–33 μm at apex, 88–190 × 13–29 μm at midleaf, and 45–221 × 20–39 μm toward insertion, due to the very wide cells, cell areolation clearly loose; decurrencies of 4–5 rows of rounded, rounded-rectangular, inflated cells, 40–90 × 22–48 μm, forming distinct, quite long auricles, 300–750 μm long (Fig. 4). Autoicous. Sporophytes abundant; setae dark brown at base, yellowish-orange at apex, twisted when dry, 1.8–2.3 cm long; the capsules more or less erect, 700–950 × 280–350 μm; operculum short and rostellate; peristome double, well developed, 450–500 μm long; exostome teeth trabeculate at the ventral side.

##### Etymology.

The present species is named in honor of Wilfred “Wilf” Borden Schofield (1927–2008), who spent decades studying northern regions of North America, including the Aleutian Islands, and who on July 19, 1996, collected the specimen (No. *106119*), chosen here as the holotype of *Plagiotheciumschofieldii*. According to Stephen Talbot (pers. comm.), Schofield recognized this plant as distinct in the field.

##### Distribution and ecology.

*Plagiotheciumschofieldii* so far has only been recorded from Adak Island, Attu Island and Simeonof Island in Alaska. In this area it has been recorded on wetlands and hills, wet cliff chimney, open, moist crevice of a cliff, shaded face of hole on slope, shaded humid outcrop, along creek and adjacent slope, near saddle between hills and near base of mountain.

##### Additional specimens examined.

U.S.A. Alaska: Adak Island, Finger Bay, along creek and adjacent slope, open, moist, crevice of cliff, 15–30 Jun 1975, *D. K. Smith 3864* (NY02589541); Attu Island, near Jaemin Pass, slopes of Ribson Ridge, shaded face of hole on slope, 52°53'N, 173°10' W, 10 Aug 2000, *W. B. Schofield* & *S. S. Talbot 115646*, UBC ACC# B185126; Shumagin Islands, Simeonof Islands, near base of larger mountain, N. side, 54°55'N, 159°15' W, shaded humid outcrop, 17 Jul 1995, *W. B. Schofield*, *S. S. Talbot* & *G. Argus 104056*, ACC# B159650 (MO5140205); wetlands and Hill 624, 54°55'N, 159°15'W, seepy cliff chimney, 7 Jul 1996, *W. B. Schofield 105769*, ACC# B161483 (MO5148015).

## Supplementary Material

XML Treatment for Plagiothecium schofieldii
